# Real-Time Panoramic Surveillance Video Stitching Method for Complex Industrial Environments

**DOI:** 10.3390/s26010186

**Published:** 2025-12-26

**Authors:** Jiuteng Zhu, Jianyu Guo, Kailun Ding, Gening Wang, Youxuan Zhou, Wenhong Li

**Affiliations:** College of Ocean Science, Shandong University of Science and Technology, Qingdao 266590, China; 202483190069@sdust.edu.cn (J.Z.);

**Keywords:** attention mechanisms, image registration, industrial surveillance, motion-aware seam optimization, real-time panoramic video stitching

## Abstract

In complex industrial environments, surveillance videos often exhibit large parallax, low illumination, low texture, and low overlap rate, making it difficult to extract reliable image feature points and consequently leading to video suboptimal stitching performance. To address these challenges, this study proposes a real-time panoramic surveillance video stitching method specifically designed for complex industrial scenarios. In the image registration stage, the Efficient Channel Attention (ECA) and Channel Attention (CA) modules are integrated with ResNet to enhance the feature extraction layers of the UDIS algorithm, thereby improving feature extraction and matching accuracy. A loss function incorporating similarity loss $L_{sim}$ and smoothness loss $L_{smooth}$ is designed to optimize registration errors. In the image fusion stage, gradient terms and motion terms are introduced for improving the energy function of the optimal seam line, enabling the optimal seam line to avoid moving objects in overlapping regions and thus achieve video stitching. Experimental validation is conducted by comparing the proposed image registration method with SIFT + RANSAC, UDIS, UDIS++, and NIS, and the proposed image fusion method with weighted average fusion, dynamic programming, and graph cut. The results show that, in image registration experiments, the proposed method achieves RMSE, PSNR, and SSIM values of 1.965, 25.338, and 0.8366, respectively. In image fusion experiments, the seam transition is smoother and effectively avoids moving objects, significantly improving the visual quality of the stitched videos. Moreover, the real-time stitching frame rate reaches 23 fps, meeting the real-time requirements of industrial surveillance applications.

## 1. Introduction

With the rapid advancement of industrial automation and computer graphics, video stitching has become a key enabling technology in various application domains, including video surveillance, autonomous driving, and virtual reality [1,2]. The core of this technology is to apply stitching algorithms to multiple video streams with overlapping regions in order to achieve seamless real-time stitching, eliminate overlapping areas, and generate a single wide-angle, large field-of-view video [3,4]. As an extension of image stitching technology, video stitching technology primarily relies on two critical stages—image registration and image fusion—both of which directly impact the quality of the final stitched output [5,6].

Image registration [7] aims to estimate the homography transformation between two images based on their extracted features [8], thereby aligning the overlapping regions of the images to be stitched. The quality of image registration directly affects the visual quality of the stitched image [9]. Traditional image registration methods are mainly based on feature point matching, but their performance is often unsatisfactory in complex industrial environments [10]. In recent years, with the development of deep learning, convolutional neural networks (CNNs) have been increasingly adopted in the field of image stitching due to their superior capability in feature extraction [11]. Zhang et al. [12] enhanced an existing network by introducing image masks, thereby improving registration performance. Song et al. [13] designed an end-to-end image stitching network based on multi-homography estimation for specific camera configurations, but its generalization ability was limited. Nie et al. [14] proposed an unsupervised learning–based image stitching framework that incorporated a low-resolution deformation branch and a high-resolution refinement branch to eliminate ghosting after initial registration; However, the network structure of the low-resolution deformation branch is too simple, and the image registration performance still needs improvement [15]. J. Ni et al. [16] proposed a fast unsupervised image stitching model that removes redundant and unnecessary sampling and convolution operations in the network, simplifying the model to achieve accelerated registration.

Image fusion [17] aims to eliminate ghosting, misalignment, and other artifacts that may appear in the overlapping regions after image registration, while optimizing the stitching seam to achieve superior visual quality [18]. Zhang J et al. [19] proposed an image fusion algorithm that searches for the optimal seam using dynamic programming, where an optimal region is selected to determine the optimal seam line. Nevertheless, the algorithm has relatively high computational complexity, and its real-time performance still requires improvement [20].

To address the issues with the aforementioned image registration methods in complex industrial scenarios, as well as the problems of image fragmentation and low real-time performance in image fusion methods when stitching video images containing moving objects, this paper proposes a real-time panoramic surveillance video stitching method suitable for complex industrial scenarios. In terms of image registration, this method abandons the traditional feature point extraction and matching approach and instead uses an unsupervised learning-based homography estimation network to perform image registration. In terms of image fusion, an improved energy function-based graph cutting method is employed to search for the optimal seam line for video image stitching. When a moving object is detected crossing the seam line region, the seam line is promptly updated to avoid the moving object, thereby enhancing fusion quality and real-time performance.

## 2. Related Work

Image and video stitching have been studied for many years. Early work is mainly based on hand-crafted features and geometric models. SIFT features together with RANSAC homography estimation [21] are often used to align overlapping images, and the final panorama is obtained by global warping or locally adaptive warps. To reduce visible seams, many methods search for an optimal path in the overlapping region or use graph cuts for image fusion. These techniques work well in many outdoor scenes, but they rely heavily on repeatable features and sufficient texture. In low-texture or low-illumination industrial environments, it is difficult to detect stable features and the performance of such pipelines quickly degrades.

In the last few years, deep learning has been introduced into image stitching. The Unsupervised Deep Image Stitching framework (UDIS and UDIS++) [14,22] learns homography estimation and image reconstruction directly from image pairs without labelled ground truth, which improves robustness under appearance changes and moderate parallax. Implicit Neural Image Stitching (NIS) [23] further represents the panorama in an implicit or frequency domain in order to recover high-frequency details and handle illumination inconsistency. Several other works add attention modules or multi-scale feature extraction to convolutional backbones so that the homography network can focus on more relevant regions. However, most of these methods are still designed for single-image stitching on general benchmarks, and they do not consider real-time constraints or the specific difficulties of industrial surveillance videos.

The recent SC-AOF method [24] combines a sliding-camera projection model with asymmetric optical flow in the blending stage to reduce perspective deformation and parallax artefacts in the stitched images. A related semi-supervised image stitching approach for unstructured camera arrays [25] uses the SandFall representation and a deep model to jointly stitch many views with higher efficiency than traditional pairwise pipelines. For industrial scenarios, Liu et al. [26] design an image stitching method for CMOS grayscale cameras under complex lighting conditions by combining a hybrid feature extraction network with plane-based constraints. There are also real-time video stitching frameworks optimized for embedded or DSP-based hardware [27,28], which mainly accelerate classical registration and blending modules for panoramic surveillance. In addition, several works on panoramic video stitching and surround-view generation [29,30] focus on global motion estimation and temporally coherent blending for automotive or general outdoor scenes.Compared with these approaches, our work focuses on real-time panoramic surveillance under challenging conditions such as low illumination, low texture, large parallax and moving objects (see Section 4). We propose an attention-enhanced unsupervised homography estimation network together with a motion-aware seam-updating fusion strategy, and demonstrate its advantages through quantitative evaluations and visual comparisons.

## 3. Methodology

### 3.1. Overall System Workflow

**Figure 1:**
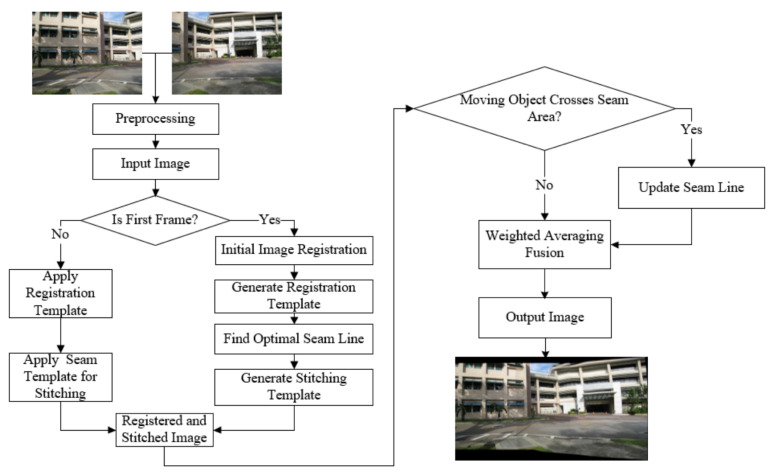
Workflow of the proposed real-time panoramic surveillance video stitching method.

The proposed real-time panoramic surveillance video stitching method for complex industrial environments consists of two primary components: video image registration based on an improved unsupervised learning framework, and video image fusion using an enhanced graph cut method for optimal seam line. The overall workflow of the proposed approach is illustrated in Figure 1.

The real-time video stitching method proposed in this paper first acquires video images captured by two surveillance cameras and performs preprocessing on them. The preprocessing steps include noise reduction and contrast enhancement to improve the accuracy and efficiency of subsequent processing. Next, The system then determines whether the current input frame is the first frame in the sequence.

- For the first frame, image registration and the search for the optimal seam line are performed, and the registration parameters and optimal seam line are saved as a template for use in subsequent frame processing.
- For non-first frames, the previously saved registration parameters and optimal seam line template are directly applied to stitch the images, resulting in registered images.

Subsequently, the registered images are analyzed to determine if any moving objects have crossed the seam line area.

- If a moving object is detected crossing the seam line, the seam line is recalculated and updated to avoid parallax artifacts in the final panoramic image.
- If no moving objects are detected, the images are directly processed using weighted average fusion to eliminate discontinuities and artifacts in overlapping regions.

**Figure 2:**
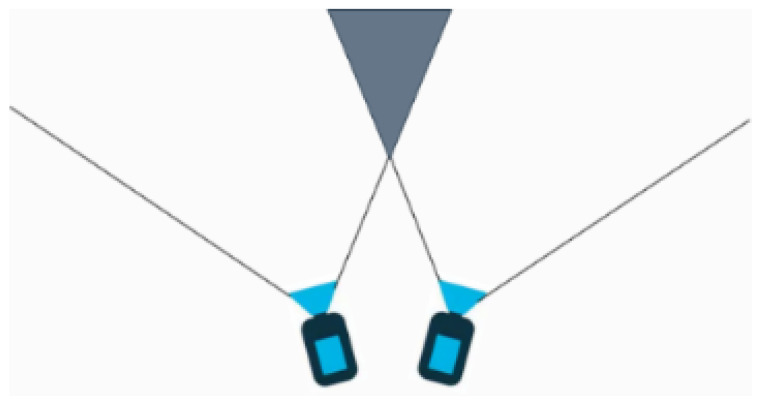
Deployment scheme of surveillance cameras. (Updated.)

Finally, the output image is aligned and free of parallax artifacts, providing a seamless panoramic surveillance video. The proposed framework is scalable to multi-camera configurations by sequentially applying pairwise registration and fusion. In this work, we demonstrate the system primarily using dual-camera pairs for algorithmic validation, and extend it to a three-camera setup for the real-world industrial experiment (see Section 4.2.2). Figure 2 illustrates a representative deployment scheme with overlapping fields of view.

### 3.2. Attention-Enhanced Unsupervised Homography Estimation for Image Registration

#### 3.2.1. Network Architecture for Unsupervised Homography Estimation

**Figure 3:**
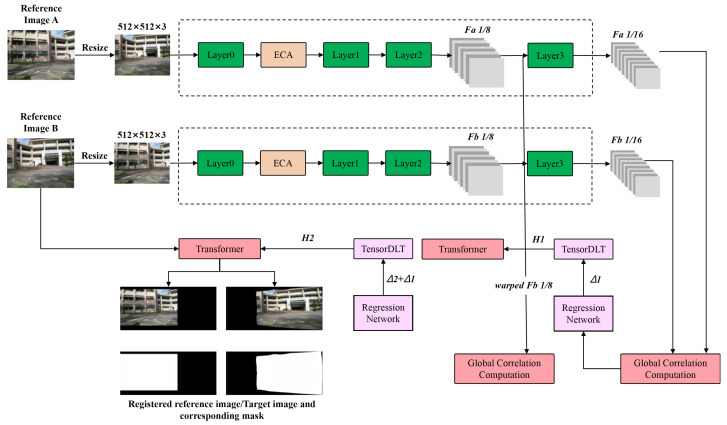
Architecture of the proposed improved unsupervised homography estimation network.

The architecture of the improved unsupervised homography estimation network is shown in Figure 3. The network comprises a feature extraction module, a global correlation computation layer, a regression network, a TensorDLT layer, and a Transformer-based warping module.

The goal of the proposed network is to accurately align a reference image $I^{A}$ with a target image $I^{B}$. The processing pipeline is summarized as follows:

- The two input images are first resized to 512×512 pixels to standardize the spatial resolution for subsequent processing.
- Both images are then fed into a Siamese (shared-weight) feature extractor, which produces a two-level feature pyramid with 1/8-resolution feature maps $F_{a}^{1/8},F_{b}^{1/8}$ and 1/16-resolution feature maps $F_{a}^{1/16},F_{b}^{1/16}$.
- At the coarse scale, $F_{a}^{1/16}$ and $F_{b}^{1/16}$ are passed to the global correlation layer to construct a dense correlation volume by computing cross-correlations between local feature blocks. This correlation volume is flattened and input to the regression network to predict eight corner displacements $\Delta_{1}$, which are converted by the TensorDLT layer into the initial homography matrix $H_{1}$.
- The initial homography $H_{1}$ is used by the Transformer-based warping module to warp the 1/8-resolution target feature map $F_{b}^{1/8}$, producing the warped feature map warped $F_{b}^{1/8}$.
- At the fine scale, the pair $\left(F_{a}^{1/8},\mathrm{warped}\;F_{b}^{1/8}\right)$ is re-fed into the global correlation layer, and the same correlation–regression–TensorDLT pipeline is applied again to obtain the refined homography matrix $H_{2}$.
- Finally, the refined homography $H_{2}$ is applied by the Transformer-based warping module to the original reference and target images, yielding the final geometrically registered image pair.

#### 3.2.2. Siamese-Based Feature Extraction Layer

**Figure 4:**
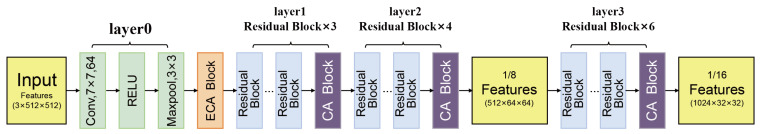
Network architecture of the feature extraction layer.

The proposed feature extraction module adopts the Siamese network paradigm, in which the input data are fed simultaneously into two identical neural network branches that share the same weights and parameters. Based on this design, a Siamese-based feature extraction layer is constructed to generate multi-scale feature maps at 1/8 and 1/16 of the original image resolution, as illustrated in Figure 4.

**Figure 5:**
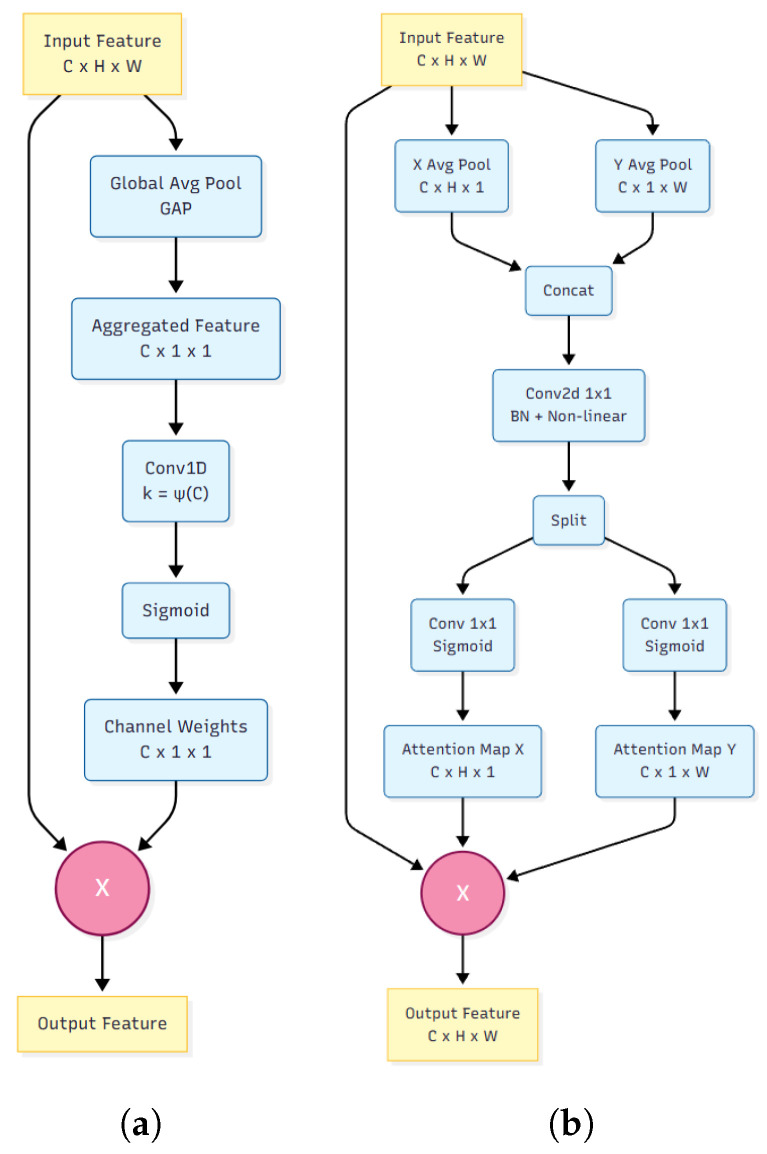
The revised architectures of the attention modules. (**a**) Efficient Channel Attention (ECA) module, which applies global average pooling followed by a 1D convolution with an adaptive kernel size k=ψ(C) to generate channel-wise weights. (**b**) Coordinate Attention (CA) module, which captures long-range spatial dependencies by decomposing channel attention into two 1D feature encoding processes along the horizontal (X-pool) and vertical (Y-pool) directions. Note: In both modules, the symbol ⊗ denotes element-wise multiplication for feature re-weighting, rather than a residual addition block.

To further enhance the feature extraction capability, two attention mechanisms are integrated into the backbone: the Efficient Channel Attention (ECA) module [31] and the Coordinate Attention (CA) module [32], whose structures are shown in Figure 5. The input image first passes through the Layer0 stage of the modified ResNet-50 backbone, which consists of a 7 × 7 convolution followed by a 3 × 3 max pooling operation. The output is then processed by the ECA module, which captures and reweights global contextual information along the channel dimension, enabling the network to focus on informative features at an early stage.

Subsequently, the image is processed by Layer1 and Layer2 of the modified ResNet-50, producing the 1/8-resolution feature maps. Passing through Layer3 yields the 1/16-resolution feature maps. To further embed positional information and capture long-range spatial dependencies, a CA module is appended after each residual block of the ResNet-50 backbone, enriching the feature representation for the subsequent image registration task.

#### 3.2.3. Loss Function

To optimize the network for precise alignment and geometric consistency, we design the total loss $L_{w}$ as a weighted sum of a similarity loss $L_{\mathrm{sim}}$ and a smoothness loss $L_{\mathrm{smooth}}$:

(1) $$L_{w}=L_{\mathrm{sim}}+\lambda L_{\mathrm{smooth}},$$

where λ is a weighting parameter used to balance the two terms. In all our experiments, we set the weighting hyperparameter λ=10.

(1) Similarity Loss ($L_{\mathrm{sim}}$). The similarity loss $L_{\mathrm{sim}}$ is designed to minimize the photometric difference between the aligned images in the overlapping region. Let $I^{A}$ and $I^{B}$ denote the reference and target images, respectively, and let H(·) be the warping operation using the estimated deformation field. We denote by *M* a binary mask with the same spatial size as $I^{A}$, where M(p)=1 if pixel *p* lies in the valid overlapping region and M(p)=0 otherwise. Instead of using a complex cross-correlation measure, we adopt the standard element-wise $L_{1}$ distance to enforce pixel-wise consistency:

(2) $$L_{\mathrm{sim}}=∥M⊙\left(H\left(I^{A}\right)-I^{B}\right){∥}_{1},$$

where ⊙ denotes element-wise multiplication and ${∥\cdot ∥}_{1}$ denotes the mean absolute error (MAE) over all pixels. This formulation is consistent with the overlap photometric loss used in our implementation.

(2) Smoothness Loss ($L_{\mathrm{smooth}}$). To prevent structural distortion and mesh folding in non-overlapping regions, we introduce a geometric smoothness loss $L_{\mathrm{smooth}}$ on the estimated deformation field. Following the grid-based regularization strategy commonly used in recent registration networks [14], this term penalizes abrupt changes of mesh edge directions and spacing so that neighbouring grids deform coherently without fold-overs. We explicitly formulate $L_{\mathrm{smooth}}$ as the sum of an inter-grid term and an intra-grid term:
  (3) $$L_{\mathrm{smooth}}=L_{\mathrm{inter}}+L_{\mathrm{intra}}.$$

The intra-grid constraint $L_{\mathrm{intra}}$ restricts the magnitude of local grid deformations to avoid excessive stretching and overlapping:

(4) $$L_{\mathrm{intra}}=\sum_{\vec{e}\in E}\mathrm{ReLU}\left(∥\vec{e}∥-\eta \right),$$

where E denotes the set of mesh edges, $∥\vec{e}∥$ is the length of edge $\vec{e}$, and η is a relaxation threshold controlling the maximum allowed edge length.

The inter-grid constraint $L_{\mathrm{inter}}$ preserves the collinearity of neighbouring edges to maintain structural rigidity of the mesh:

(5) $$L_{\mathrm{inter}}=\sum_{({\vec{e}}_{1},{\vec{e}}_{2})\in P}\left(1-\frac{{\vec{e}}_{1}\cdot {\vec{e}}_{2}}{∥{\vec{e}}_{1}∥∥{\vec{e}}_{2}∥}\right),$$

where P is the set of adjacent edge pairs along the horizontal and vertical grid directions. By penalizing deviations from parallelism, $L_{\mathrm{inter}}$ encourages neighbouring grid cells to deform in a coherent manner without abrupt bending.

In all our experiments, we set the weighting hyperparameter λ=10 to emphasize the geometric regularization $L_{\mathrm{smooth}}$ while maintaining accurate photometric alignment in the overlapping regions.

### 3.3. Motion-Aware Graph Cut Fusion for Optimal Seam Selection

In panoramic video stitching, determining an optimal seam is crucial for preventing ghosting and preserving structural consistency, especially in industrial scenes that contain dynamic objects, parallax, and complex textures. Traditional graph-cut–based seam selection methods rely solely on color or gradient cues and often fail when the two camera views differ significantly or contain independently moving regions. To address these challenges, our method introduces a motion-aware graph cut fusion strategy that incorporates both spatial cues (color, smoothness, gradient) and temporal motion information. This enables more stable and visually coherent seam placement across frames, ensuring robustness under real-world industrial surveillance conditions.

#### 3.3.1. Principle of Graph Cut–Based Seam Search

In the graph cut framework, an image is represented as a graph in which the nodes correspond to pixels and the edges encode the neighborhood relationships between them. By formulating an energy function and assigning appropriate weights to the nodes and edges, the optimal stitching seam can be determined by computing the minimum cut on the weighted graph, which partitions it into two disjoint sets (foreground and background). The resulting minimum cut path corresponds to the optimal seam line, and it can be efficiently obtained using the max–flow/min–cut algorithm.

Consequently, the quality of the seam is determined by the formulation of the energy function, specifically the assignment of weights to the nodes and edges. An appropriately designed energy function guides the seam toward visually inconspicuous areas while steering it away from salient structures and moving objects, which is essential for achieving high-quality fusion in panoramic video stitching.

#### 3.3.2. Energy Function Formulation

To compute the optimal seam in the overlapping region between the two registered images, we formulate a binary labeling problem. Let Ω denote the set of pixels in the overlapping region between the two images. For each pixel p∈Ω, we assign a binary label $l_{p}\in \left\{0,1\right\}$, where $l_{p}=0$ means that the pixel value at *p* is taken from the first image and $l_{p}=1$ means that it is taken from the second image. We write $l=\{l_{p}\}$ for the collection of all labels. Let N(Ω) denote the set of neighbouring pixel pairs (p,q) inside Ω. For each subsequent frame, $M_{\Omega }\subset \Omega$ denotes the detected moving-object region in the overlap.

The energy of a labeling *l* is composed of a smoothness term and a gradient term, together with two hard constraints that restrict the feasible seam domain:

- smoothness term $E_{s}\left(p,q,l_{p},l_{q}\right),$
- gradient term $E_{g}\left(p,q,l_{p},l_{q}\right),$
- data-domain constraint $E_{d}\left(p,l_{p}\right),$
- motion-region constraint $E_{m}\left(p,l_{p}\right).$

For the first frame, only the data-domain constraint is imposed and the seam is allowed to pass anywhere within Ω. Consequently, the energy to be minimized comprises the smoothness term and the gradient term over Ω:

(6) $$E_{\mathrm{first}}\left(l\right)=\alpha \sum_{\left(p,q)\in N(\Omega \right)}E_{s}\left(p,q,l_{p},l_{q}\right)+\beta \sum_{\left(p,q)\in N(\Omega \right)}E_{g}\left(p,q,l_{p},l_{q}\right),$$

where α and β are the parameters that balance the weights of the smoothness and gradient terms. Since $E_{s}$ and $E_{g}$ play similar roles of penalizing noticeable seams, we assign equal importance to both terms and simply set α=β=1 in all experiments. The data-domain constraint $E_{d}$ is implemented by constructing the graph only over Ω (only pixels in Ω are included as nodes), so it acts as a 0/+*∞* indicator of the valid domain. As a result, its contribution to the total energy is constant for all feasible labelings and does not appear explicitly in Equation (6).

For subsequent frames, the detected moving-object region $M_{\Omega }$ is excluded from the graph so that the seam is searched only in the remaining background region Ω∖$M_{\Omega }$. The corresponding energy is defined as

(7) $$E_{\mathrm{sub}}\left(l\right)=\alpha \sum_{\left(p,q\right)\in N\left(\Omega \setminus M_{\Omega }\right)}E_{s}\left(p,q,l_{p},l_{q}\right)+\beta \sum_{\left(p,q\right)\in N\left(\Omega \setminus M_{\Omega }\right)}E_{g}\left(p,q,l_{p},l_{q}\right).$$

Here the motion constraint $E_{m}$ changes the feasible seam domain by removing $M_{\Omega }$ from the graph. Similar to the data-domain constraint, it is implemented as a 0/+*∞* indicator and therefore contributes only a constant value to the total energy for all admissible labelings, while effectively forcing the optimal seam to avoid moving objects.

1. Data-domain constraint

The data-domain constraint ensures that the seam stays inside the overlapping region Ω. It can be written as the following indicator function:
  (8) $$\begin{array}{c} E_{d}\left(p,l_{p}\right)=\left\{\begin{array}{cc} 0, & p\in \Omega ,\\ +\infty , & p\notin \Omega . \end{array}\right. \end{array}$$
  In practice, we enforce this constraint by constructing the graph only on Ω, so $E_{d}$ does not explicitly appear in Equations (6) and (7).

2. Smoothness term

The smoothness term guides the seam to pass through visually homogeneous regions by penalizing label discontinuities between neighbouring pixels:
  (9) $$E_{s}\left(p,q,l_{p},l_{q}\right)=\left|l_{p}-l_{q}\right|\left(I_{d}\left(p\right)+I_{d}\left(q\right)\right).$$
  Here, $I_{d}\left(\cdot \right)$ measures the color difference between the two input images at a given pixel location. Let $I_{0}$ and $I_{1}$ denote the intensity functions of the first and second images, respectively. The color-difference measure is defined as
  (10) $$I_{d}\left(p\right)=∥I_{0}\left(p\right)-I_{1}\left(p\right){∥}_{2}^{2}.$$
  Thus, larger appearance differences at (p,q) lead to higher penalties when the labels $l_{p}$ and $l_{q}$ are different, encouraging the seam to pass through visually smooth regions.

3. Gradient term

The gradient term $E_{g}\left(p,q,l_{p},l_{q}\right)$ is intended to distinguish foreground objects from the background according to the geometric structure of the scene. By incorporating gradient information between pixels, it enhances the edge-detection capability of the seam search and reduces the likelihood that the optimal seam passes through salient objects (e.g., buildings), preferring background regions instead. The gradient term is defined as
  (11) $$E_{g}\left(p,q,l_{p},l_{q}\right)=\left|l_{p}-l_{q}\right|\left(W(p)+W(q)\right),$$
  where W(·) is an edge-weight map computed from the image gradients:
  (12) $$W\left(p\right)=\sigma \left(W_{x}\left(p\right)+W_{y}\left(p\right)\right),$$
  with σ(·) denoting the sigmoid function. Let $I_{0}$ and $I_{1}$ denote the intensity functions of the first and second images, respectively. For a pixel *p* with label $l_{p}\in \left\{0,1\right\}$, we write $I_{l_{p}}$ for the corresponding intensity function, i.e., $I_{l_{p}}=I_{0}$ if $l_{p}=0$ and $I_{l_{p}}=I_{1}$ if $l_{p}=1$. Using this notation, the horizontal and vertical gradient energies are given by
  (13) $$\begin{array}{cc} W_{x}\left(p\right) & ={\left([S_{x}*\left(I_{l_{p}}\left(\cdot \right)-I_{l_{q}}\left(\cdot \right)\right)]\left(p\right)\right)}^{2}, \end{array}$$
  (14) $$\begin{array}{cc} W_{y}\left(p\right) & ={\left([S_{y}*\left(I_{l_{p}}\left(\cdot \right)-I_{l_{q}}\left(\cdot \right)\right)]\left(p\right)\right)}^{2}, \end{array}$$
  where ∗ denotes convolution, and $S_{x}$ and $S_{y}$ are the Sobel operators in the horizontal and vertical directions, respectively, with kernels
  (15) $$S_{x}=\left[\begin{array}{ccc} -2 & 0 & 2\\ -1 & 0 & 1\\ -2 & 0 & 2 \end{array}\right],S_{y}=\left[\begin{array}{ccc} -2 & -1 & -2\\ 0 & 0 & 0\\ 2 & 1 & 2 \end{array}\right].$$

4. Motion-region constraint

The motion-region constraint separates moving foreground objects from the static background so that the optimal seam prefers to traverse background regions. Let $M_{\Omega }\subset \Omega$ denote the moving-object region detected in the overlapping area. The corresponding indicator function is defined as
  (16) $$\begin{array}{c} E_{m}\left(p,l_{p}\right)=\left\{\begin{array}{cc} 0, & p\notin M_{\Omega },\\ +\infty , & p\in M_{\Omega }. \end{array}\right. \end{array}$$
  In other words, any labeling whose seam passes through $M_{\Omega }$ incurs an infinite energy and is therefore infeasible. When $E_{m}$ is incorporated into the energy formulation, this is equivalent to restricting the optimization domain from Ω to Ω∖$M_{\Omega }$, as reflected in Equation (7).

**Figure 6:**
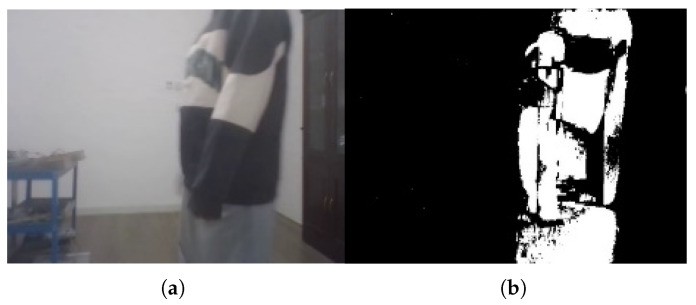
Motion object detection results: (**a**) original image; (**b**) detected moving object regions.

The detection results are shown in Figure 6, where (a) represents the original image and (b) shows the detected moving object regions.

## 4. Experimental Results and Analysis

To evaluate the effectiveness and practicality of the proposed panoramic video stitching method, this section presents a series of experiments on both a public synthetic dataset and a composite dataset that includes real industrial scenes. We first describe the experimental environment and datasets, and then assess the registration performance of our network in comparison with several representative traditional and learning-based methods, using RMSE, PSNR, SSIM, and runtime as evaluation metrics. Subsequently, we analyze the visual stitching quality of different methods under four typical scenarios—large parallax, low illumination, low texture, and low overlap. Finally, we investigate the behaviour of the proposed fusion strategy in dynamic scenes and its real-time processing performance. These experiments together demonstrate that the proposed method can achieve accurate and efficient panoramic video stitching under challenging surveillance conditions.

### 4.1. Image Registration Experiments and Analysis

#### 4.1.1. Experimental Setup and Training Procedure

The proposed image registration network was tested under the following hardware and software environment: a 13th-generation Intel(R) Core(TM) i7-13700 CPU (Intel, Santa Clara, CA, USA), an NVIDIA GeForce RTX 3060 GPU (Nvidia, Santa Clara, CA, USA), and the Windows 10 operating system. The network was implemented using the PyTorch (version 1.7.1) deep learning framework, and we trained the network using Adam optimizer with an initial learning rate of $1\times 10^{-4}$. The Adam hyper-parameters were fixed to $\beta_{1}=0.9$, $\beta_{2}=0.999$ and $\epsilon =10^{-8}$. The learning rate was exponentially decayed by a factor of 0.97 at the end of each epoch using an ExponentialLR scheduler.

**Figure 7:**
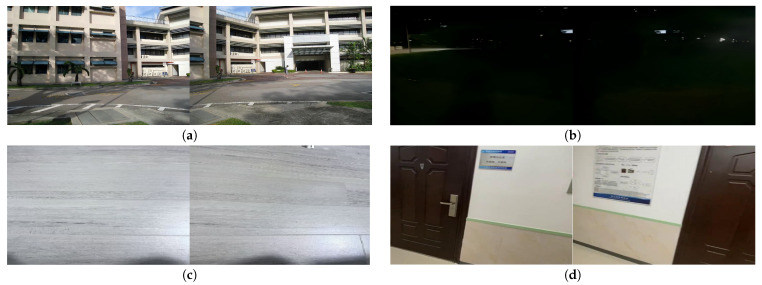
(Updated) Examples of challenging scenarios in the synthetic dataset: (**a**) large parallax, (**b**) low illumination, (**c**) low texture, and (**d**) low overlap. Each group contains a pair of images representing the same condition.

To train and evaluate the registration network, two datasets were utilized. The first is the Warped MS-COCO dataset [33], which provides a large number of synthetic image pairs with known homographies and is widely used for pre-training homography estimation networks. The second is a composite dataset constructed by combining the UDIS-D dataset [14] and a set of real-world scene images collected for this study. The composite dataset features image pairs with varying degrees of parallax, illumination, texture richness, and overlapping regions, as illustrated in Figure 7. Specifically, the training set contains 11,440 image pairs, and the test set contains 1206 image pairs.

The training was conducted in two stages. First, the network was trained for 100 epochs on the Warped MS-COCO dataset to learn basic image registration features. Then, a fine-tuning stage of 50 epochs was performed on the composite dataset to improve the network’s ability to generalize to complex, real-world industrial scenes.

#### 4.1.2. Registration Performance Evaluation

To comprehensively evaluate the performance of the proposed registration network, we compare it with several baselines, including the identity transformation ($I_{3\times 3}$), the traditional feature-based method SIFT + RANSAC [14], and state-of-the-art unsupervised deep learning methods: UDIS [14], UDIS++ [22], and NIS [23].

Following the standard evaluation protocol established in [14,22], we categorize the test samples into three subsets based on the overlap rate between the input image pairs:

- 0–30%: Low overlap rate, typically corresponding to large-parallax scenes that are most challenging for registration.
- 30–60%: Medium overlap rate representing standard registration scenarios.
- 60–100%: High overlap rate where images share most of the content.

For quantitative evaluation, we report the average metrics on each subset as well as the overall dataset. On the Warped MS-COCO dataset, we use the Root Mean Square Error (RMSE) to quantify registration accuracy. On the synthetic dataset, we employ the Structural Similarity Index Measure (SSIM) and Peak Signal-to-Noise Ratio (PSNR) to assess the perceptual quality of the aligned images. Additionally, to evaluate real-time performance in industrial surveillance scenarios, we compare the average runtime of each method for registering a single image pair.

(1) Root Mean Square Error (RMSE)

The Root Mean Square Error (RMSE) is a measure that reflects the magnitude of the average error between the predicted value and the ground truth. A smaller RMSE value indicates greater accuracy in the registration or measurement technique.

As analyzed in comparison with Table 1, the RMSE of our proposed network shows a significant improvement compared to traditional and existing unsupervised methods:

- The RMSE was reduced by approximately 77.06% compared to the SIFT + RANSAC method.
- The RMSE was reduced by approximately 7.1% compared to the UDIS algorithm.
- The RMSE was reduced by approximately 2.19% compared to the NIS algorithm, although it marginally underperforms the UDIS++ algorithm by 6.51%.

(2) Peak Signal-to-Noise Ratio (PSNR)

PSNR is an objective metric, measured in decibels (dB), used to quantify the perceptual quality of images and videos, reflecting the influence of noise introduced during compression or processing on the original signal. Higher PSNR values indicate better image preservation.

As analyzed in comparison with Table 2, the PSNR of our proposed network demonstrates clear enhancements:

- PSNR increased by 4.367 dB compared to the SIFT + RANSAC method.
- PSNR increased by 1.951 dB compared to the UDIS algorithm.
- PSNR increased by 0.973 dB compared to the NIS algorithm, although it marginally underperforms the UDIS++ algorithm by 1.12 dB.

(3) Structural Similarity Index Measure (SSIM)

The Structural Similarity Index Measure (SSIM) is an objective metric used to assess the perceived similarity between two images based on three factors: luminance, contrast, and structure. Values closer to 1 indicate higher structural similarity and better image quality.

As analyzed in comparison with Table 3, the SSIM value of our proposed network shows consistent gains:

- SSIM increased by 0.1669 compared to the SIFT + RANSAC method.
- SSIM increased by 0.0724 compared to the UDIS algorithm.
- SSIM increased by 0.0143 compared to the NIS algorithm, although it marginally underperforms the UDIS++ algorithm by 0.0297.

(4) Runtime and Overall Conclusion

As derived from the comparison results in Table 4, our improved registration network achieves the lowest computational cost. Despite the overall registration quality being marginally lower than the best-performing UDIS++ algorithm, the improved network requires only 53 ms to register a single image pair, which better meets the real-time requirements of industrial workshop application scenarios.

To ensure the reliability and verifiability of our real-time claims in Table 4 and Table 5, all latency measurements were conducted on a desktop machine equipped with an NVIDIA GeForce RTX 3060 GPU (Nvidia, Santa Clara, CA, USA) and an Intel Core i7 CPU (Intel, Santa Clara, CA, USA). Unless otherwise stated, the input resolution for all methods is fixed to 512×512 pixels, which matches the typical size of the cropped overlapping region processed in our industrial monitoring scenario.

For each algorithm, we follow a standard low-latency evaluation protocol. Specifically, we first run 100 warm-up iterations to stabilize the GPU cache and memory allocation; these runs are discarded. We then record the wall-clock inference time over 1000 repeated measurements and report the average per-image-pair runtime in milliseconds. The proposed method achieves an average registration time of 53 ms per image pair under this setting, which is sufficient for near real-time processing in our target application. The comprehensive quantitative comparison demonstrates that the improved registration network simultaneously enhances the visual quality of the aligned image while reducing computational overhead.

**Figure 8:**
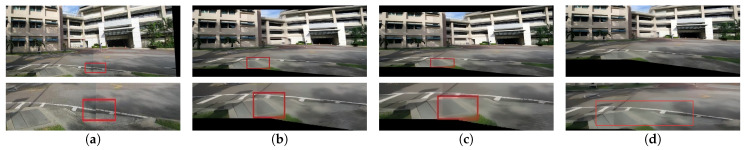
(Updated) Comparison in large parallax scenarios. (**a**) SIFT + RANSAC. (**b**) UDIS++. (**c**) NIS. (**d**) the proposed method. The red rectangles indicate representative regions along the stitching seam, and the bottom row shows the corresponding magnified views.

**Figure 9:**
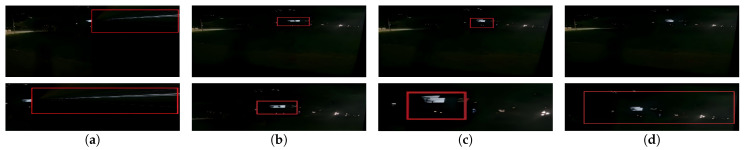
(Updated) Comparison in low illumination scenarios. (**a**) SIFT + RANSAC. (**b**) UDIS++. (**c**) NIS. (**d**) the proposed method. The red rectangles indicate representative regions along the stitching seam, and the bottom row shows the corresponding magnified views.

**Figure 10:**
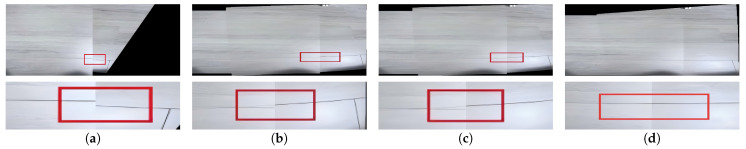
(Updated) Comparison in low-texture scenarios. (**a**) SIFT + RANSAC. (**b**) UDIS++. (**c**) NIS. (**d**) the proposed method. The red rectangles indicate representative regions along the stitching seam, and the bottom row shows the corresponding magnified views.

**Figure 11:**
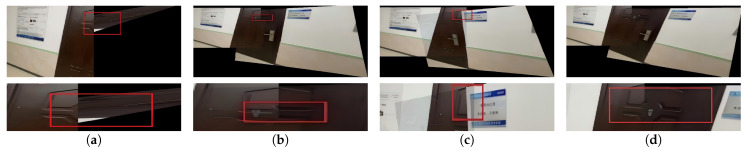
(Updated) Comparison in low-overlap scenarios. (**a**) SIFT + RANSAC. (**b**) UDIS++. (**c**) NIS. (**d**) the proposed method. The red rectangles indicate representative regions along the stitching seam, and the bottom row shows the corresponding magnified views.

To validate the effectiveness of the improved registration network in complex scenarios such as large parallax, low illumination, low texture, and low overlap rate, a comparative visual evaluation of registration results using different methods was conducted. The results are shown in Figure 8, Figure 9, Figure 10 and Figure 11 respectively.

To better inspect the local stitching quality, the red rectangles in Figure 8, Figure 9, Figure 10 and Figure 11 mark representative regions along the stitching seam for each method, and the second row shows magnified views of these regions.

Visual inspection of Figure 8, Figure 9, Figure 10 and Figure 11 reveals additional insights into the comparative performance of each method. Traditional feature-based methods like SIFT + RANSAC fail in low-illumination, low-texture, and low-overlap scenes due to difficulty in reliable keypoint extraction. Although UDIS++ and NIS perform well in most scenarios, our method demonstrates superior handling of specific challenging cases with more stable results across varied conditions.

In summary, the proposed registration network shows excellent robustness and accuracy in challenging industrial environments and outperforms traditional and existing unsupervised methods in most scenarios, making it a strong candidate for real-world panoramic video stitching systems.

### 4.2. Image Fusion Evaluation

#### 4.2.1. Fusion Performance in Dynamic Scenes

**Figure 12:**
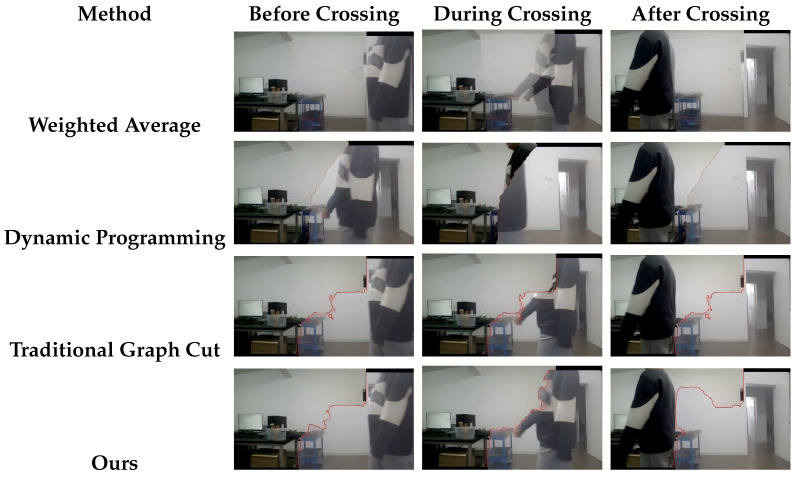
Comparative results of image fusion when objects cross the seam.

To validate the effectiveness of the proposed method in dynamically updating the seam line and avoiding moving regions when objects pass through the seam area, a comparative experiment was conducted. The experiment involved four fusion strategies: the proposed motion-aware graph cut–based method, the weighted averaging method [34], the dynamic programming–based method [35], and the traditional graph cut method [36]. For the weighted averaging baseline, no seam is used and the overlapping region is blended by simple linear weighting. For the DP and GC baselines, an optimal seam is recomputed for every frame using their originalenergy functions based on color and gradient consistency. In contrast, our method also recomputes the seam for every frame, but augments the graph-cutenergy with a motion-aware term that explicitly penalizes seams passing through moving regions.Fusion results were evaluated under three conditions: before the object crosses the seam, during the crossing, and after the object has passed through. The results are presented in Figure 12.

The analysis of Figure 12 reveals the performance of different image fusion strategies in dynamic scenarios. The weighted averaging method exhibits significant ghosting artifacts when objects pass through the overlapping region of the videos, due to its inability to handle the temporal continuity of moving objects. This leads to degraded visual quality, particularly around object boundaries.

In contrast, the dynamic programming method and the traditional graph cut method theoretically provide better seam search strategies. However, in practice, both methods fail to update the seam in response to object motion in time, resulting in visual discontinuities when the object crosses the overlapping area. These discontinuities break the visual coherence and degrade the overall stitching quality.

Unlike these methods, the proposed approach can dynamically update the seam line just before the object crosses it, effectively avoiding moving regions. This adaptive seam updating strategy significantly enhances the visual consistency of the stitched image, reducing ghosting and disconnection artifacts caused by moving objects and maintaining a smooth, natural panoramic output.

It is worth noting that, although the DP and GC seams are recomputed at every frame, they still appear almost fixed. This is because their energies rely solely on static color and gradient differences. In our surveillance videos, the global minimum of such an energy often lies along similar low-texture background regions across frames. Consequently, the per-frame optimal seams tend to stay in nearly the same location and fail to avoid moving objects, which explains the visible discontinuities in their results.

In summary, the experimental results demonstrate that the proposed fusion method outperforms weighted averaging, dynamic programming, and traditional graph cut–based methods in handling dynamic scenes. A slight distortion can still be observed around the arm in our result, mainly due to imperfect registration under large parallax and fast motion. We consider further improving the motion modeling and alignment quality in such challenging cases as future work.

#### 4.2.2. Real-World Industrial Video Stitching

**Figure 13:**
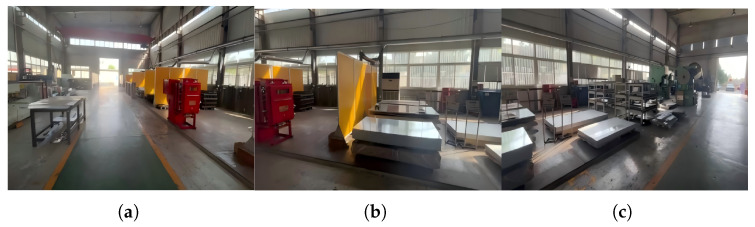
Three-channel workshop video stitching process. (**a**) Source video 1; (**b**) Source video 2; (**c**) Source video 3.

**Figure 14:**
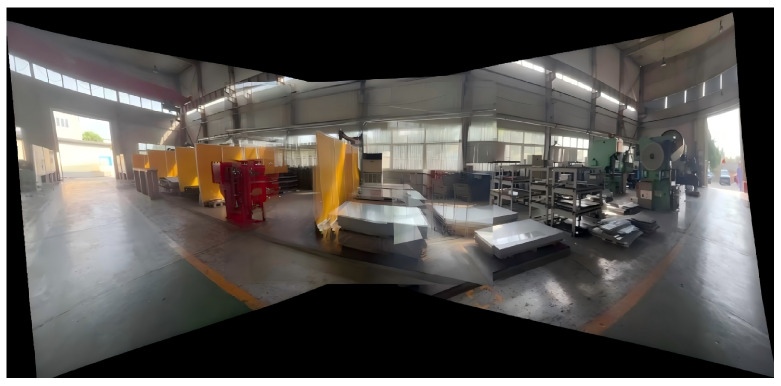
Registered video frames.

**Figure 15:**
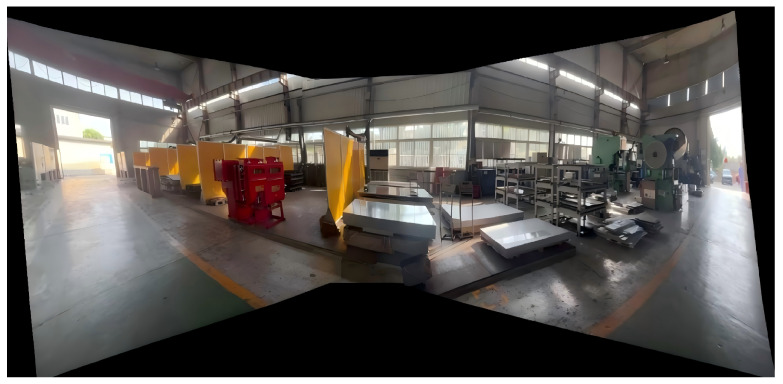
Fused video frames.

To further evaluate the real-time performance of the proposed seam-cutting and fusion method in practical scenarios, we conduct an experiment on a real industrial workshop scene. Three fixed surveillance cameras are installed along an assembly line, providing partially overlapping fields of view. Each camera outputs video streams at a resolution of 1920×1080 and a frame rate of 30 fps. The stitching process is illustrated in Figure 13, Figure 14 and Figure 15. To balance computational efficiency with alignment accuracy during real-time processing, the input frames are resized to 512×512 pixels before being fed into the registration and fusion network, which is consistent with the runtime setting reported in Table 4. The overlapping regions among the three views are similar to the deployment in our target industrial monitoring system.

For all methods, each incoming frame triplet is first registered and then fused online. The overall stitching throughput is measured as the effective output frame rate of the stitched video. Table 5 reports the average per-frame processing time of the seam search, seam update, and image fusion stages, as well as the resulting stitching frame rate (fps) for all four compared methods.

In the seam searching stage, the Weighted Averaging method employs a global blending strategy across the entire overlapping region, eliminating the need for a seam searching process (indicated as “–” in the table). the proposed method records the highest runtime (332 ms), which is higher than both Dynamic Programming (253 ms) and Traditional Graph Cut (107 ms). This increased computational cost is primarily due to the integration of our advanced motion-aware mechanism. Unlike traditional methods that only consider static color gradients, our approach additionally calculates motion gradients and detects moving objects during the search process to ensure artifact-free stitching, which inherently requires more processing time.

In the seam updating stage, similarly, the Weighted Averaging method does not involve seam updating. The proposed method (17 ms) performs comparably to the baseline methods. demonstrating that our dynamic update strategy is computationally efficient and adds negligible overhead. In the image fusion stage, the Weighted Averaging method is the slowest (35 ms) as it requires pixel-wise blending for every frame. In contrast, our method, along with Dynamic Programming and Traditional Graph Cut, achieves a more efficient fusion time (24 ms) by utilizing the pre-calculated optimal seam.

Despite the higher cost in the initial seam search, the proposed method achieves an impressive effective frame rate of 23 fps in the continuous video stitching test. This performance is on par with Dynamic Programming (23 fps) and only slightly lower than the Traditional Graph Cut (25 fps) and Weighted Averaging (28 fps). This indicates that our method successfully meets the real-time requirement (>20 fps) for industrial monitoring.

In conclusion, although the proposed method sacrifices some computational speed in the seam search stage to incorporate motion awareness, it still maintains a high overall frame rate of 23 fps. This trade-off is justified by the significant improvement in visual quality and the effective avoidance of ghosting artifacts in dynamic scenes, making it a robust solution for complex industrial surveillance applications.

## 5. Conclusions

In this paper, we propose a real-time panoramic video stitching method tailored to challenging industrial environments characterized by large parallax, low illumination, low texture, and limited overlap. The proposed approach comprises two major components: an unsupervised learning-based registration network and a motion-aware graph cut fusion strategy.

In the registration phase, a feature extraction backbone enhanced with attention mechanisms is used to improve robustness under difficult imaging conditions. The network demonstrates strong generalization capability, as evidenced by its performance on both synthetic and real-world datasets. Compared to traditional methods such as SIFT + RANSAC and existing unsupervised approaches like UDIS, UDIS++, and NIS, our method achieves higher registration accuracy and better visual quality, while also maintaining fast inference speed suitable for real-time deployment.

In the fusion stage, we introduce a motion-aware energy model into the graph cut framework to ensure temporal consistency across frames. When moving objects cross the seam line, the system dynamically updates the optimal seam to avoid cutting through salient motion regions. Comparative experiments with weighted average blending, dynamic programming, and conventional graph cuts show that our method effectively eliminates ghosting and tearing artifacts, delivering smoother and more natural visual results in dynamic scenes.

Although the proposed method achieves competitive performance, several limitations remain. First, our current implementation is built around pairwise registration and fusion, and in this paper it is validated on a dual-camera configuration for the benchmark datasets and on a three-camera surveillance setup in an industrial workshop. Extending the framework to arbitrary large-scale multi-camera systems with globally consistent optimisation across many views (e.g., loop closure and coordinated multi-seam management) is an interesting direction for future work. Second, all experiments are conducted with static surveillance cameras; handling camera motion (e.g., on mobile or drone platforms) is left for future investigation. Third, our prototype is evaluated on a desktop GPU platform (see Section 4), and deploying the system on resource-constrained edge devices would require additional optimisation and engineering effort, which is beyond the scope of this paper.

In summary, our method achieves a strong balance between accuracy and efficiency. It is well-suited for real-time panoramic stitching in industrial monitoring systems and shows great potential for future extension to more general applications, such as mobile or drone-based video stitching. Future work will explore adaptive mechanisms for camera motion and lightweight network architectures to further enhance performance and generalization.

## Figures and Tables

**Table 1:** Comparison of RMSE on the MS-COCO dataset.

Method	Root Mean Square Error (RMSE)			
	0∼30%	30∼60%	60∼100%	Average
$I_{3\times 3}$	15.13	18.32	21.53	18.647
SIFT + RANSAC	0.76	1.36	19.83	8.568
UDIS	1.28	1.57	3.15	2.115
UDIS++	1.09	1.38	2.76	1.845
NIS	1.20	1.47	3.02	2.009
Proposed Method	1.14	1.45	2.97	1.965

**Table 2:** PSNR Comparison on Synthetic Dataset.

Method	Peak Signal-to-Noise Ratio (PSNR/dB)			
	0∼30%	30∼60%	60∼100%	Average
$I_{3\times 3}$	15.62	12.53	10.45	12.625
SIFT + RANSAC	24.85	21.96	17.32	20.971
UDIS	26.91	23.78	20.45	23.387
UDIS++	31.16	26.86	22.63	26.458
NIS	28.33	25.06	21.32	24.545
Proposed Method	30.02	25.73	21.57	25.338

**Table 3:** SSIM Comparison on the Synthetic Dataset.

Method	Peak Signal-to-Noise Ratio (PSNR/dB)			
	0∼30%	30∼60%	60∼100%	Average
$I_{3\times 3}$	0.3736	0.1586	0.0658	0.1860
SIFT+RANSAC	0.8143	0.7261	0.5145	0.6679
UDIS	0.8945	0.7992	0.6357	0.7624
UDIS++	0.9573	0.8915	0.7791	0.8663
NIS	0.9037	0.8638	0.7302	0.8223
Proposed Method	0.9312	0.8728	0.7385	0.8366

**Table 4:** Comparison of Algorithm Registration Runtime.

Method	Runtime (ms)
UDIS	122
UDIS++	86
NIS	75
Proposed Method	**53**

**Table 5:** Comparison of time consumption and real-time frame rate of different methods for splicing.

Method	Algorithm Runtime (ms)			Avg. FPS
	Search	Update	Fusion	
Weighted Averaging	–	–	35	28
Dynamic Programming	253	18	24	23
Traditional Graph Cut	107	17	24	25
Proposed Method	332	17	24	23

## Data Availability

The UDIS-D dataset used in this study is publicly available at https://github.com/nie-lang/UnsupervisedDeepImageStitching (accessed on 21 October 2025). The factory dataset used in this study is not publicly available due to confidentiality agreements.
